# Reduced dynamic changes in pulmonary artery compliance during isometric handgrip exercise in patients with heart failure

**DOI:** 10.1038/s41598-024-66194-8

**Published:** 2024-07-06

**Authors:** Djawid Hashemi, Xuewen Hou, Patrick Doeblin, Jakob Weiß, Rebecca Beyer, Marthe Neye, Jennifer Erley, Paulius Bucius, Radu Tanacli, Titus Kuehne, Marcus Kelm, Moritz Blum, Frank Edelmann, Wolfgang M. Kuebler, Hans-Dirk Düngen, Andreas Schuster, Lukas Stoiber, Sebastian Kelle

**Affiliations:** 1 Department of Cardiology, Angiology and Intensive Care Medicine, Deutsches Herzzentrum der Charité, Charité – Universitätsmedizin Berlin, Augustenburger Platz 1, 13353 Berlin, Germany; 2 Charité – Universitätsmedizin Berlin, Corporate Member of Freie Universität Berlin and Humboldt-Universität zu, Berlin, Charitéplatz 1, 10117 Berlin, Germany; 3 DZHK (German Center for Cardiovascular Research), Partner Site Berlin, Berlin, Germany; 4 BIH Biomedical Innovation Academy, BIH Charité Digital Clinician Scientist Program, Berlin Institute of Health at Charité – Universitätsmedizin Berlin, Charitéplatz 1, 10117 Berlin, Germany; 5 Department of Diagnostic and Interventional Radiology and Nuclear Medicine, University Medical Center Hamburg-Eppendorf, Hamburg, Germany; 6 Department of Cardiology, Medical Academy, Lithuanian University of Health Sciences, Kaunas, Lithuania; 7 Institute of Computer-Assisted Cardiovascular Medicine, Deutsches Herzzentrum der Charité, Augustenburger Platz 1, 13353 Berlin, Germany; 8 Department of Congenital Heart Disease – Pediatric Cardiology, Deutsches Herzzentrum der Charité, Augustenburger Platz 1, 13353 Berlin, Germany; 9 Brookdale Department of Geriatrics and Palliative Medicine, Icahn School of Medicine at Mount Sinai, New York, NY USA; 10 Department of Cardiology and Pneumology, University Medical Center Göttingen, Georg-August University, Göttingen, Germany; 11 Institute of Physiology, Charité-Universitätsmedizin Berlin, Berlin, Germany; 12 DZHK (German Center for Cardiovascular Research), Partner Site Göttingen, Göttingen, Germany; 13 Royal Brompton Hospital, Guy’s and St Thomas’ National Health Service Foundation Trust, London, UK

**Keywords:** Epidemiology, Outcomes research

## Abstract

Exercise intolerance is a debilitating symptom in heart failure (HF), adversely affecting both quality of life and long-term prognosis. Emerging evidence suggests that pulmonary artery (PA) compliance may be a contributing factor. This study aims to non-invasively assess PA compliance and its dynamic properties during isometric handgrip (HG) exercise in HF patients and healthy controls, using cardiovascular magnetic resonance (CMR). We prospectively enrolled 36 subjects, comprising 17 HF patients (NYHA class II and III) and 19 healthy controls. Participants performed an HG test, and we assessed changes in PA compliance and hemodynamic flow parameters using advanced CMR techniques. We also explored the relationship between CMR-derived PA compliance metrics and established clinical indicators, ensuring the validity of our findings through intra- and interobserver agreements. HF patients had significantly lower resting PA compliance compared to controls (28.9% vs. 50.1%, *p* < 0.01). During HG exercise, HF patients exhibited a dampened adaptability in PA compliance. Hemodynamic responses, including heart rate and blood pressure, were not significantly different between the groups. Further analyses revealed a significant correlation between changes in PA compliance and functional capacity, and an inverse relationship with NYHA class. Our study demonstrates a marked difference in PA vascular responses during HG exercise between HF patients and healthy controls. The compromised adaptability in PA compliance in HF patients is correlated with diminished functional capacity. These findings have significant clinical implications and may guide future interventional strategies in HF management.

## Introduction

Heart failure (HF) is a global health problem and a major cause of morbidity and mortality^1^. Despite advances in treatment strategies, the prognosis of patients with HF remains poor and is heterogeneous across the population^2^. The interaction of cardiac and vascular function is a key determinant of cardiovascular performance, and understanding the pathophysiology of HF is crucial for developing effective therapies^3,4^. Pulmonary hypertension (PH) is highly prevalent among patients with HF and is associated with worse outcomes^5^. Previously, much focus has been given to pulmonary artery (PA) stiffness as a manifestation of PH; however, its counter-concept, PA compliance, remains less understood, particularly in a dynamic context. PA stiffness is a manifestation of PH, but its prognostic role in HF remains to be fully understood^3,6–8^. In our previous study, we found that PA stiffness at rest was significantly increased in HF patients and was associated with higher NT-proBNP levels and reduced functional status^9^. This highlighted the importance of vascular properties in HF and set the stage for investigating PA compliance. Understanding PA compliance could provide further insights into dynamic vascular changes during exercise, with significant clinical implications for HF management. PA compliance may be understood as comprising both dynamic and static elements. The dynamic aspect pertains to alterations in PA compliance in response to stress or exercise, whereas the static aspect involves the inherent levels of PA compliance under basal conditions. Both elements could be critical for the pathology and clinical management of HF. Notably, HF patients represent a heterogeneous population with varying etiologies and clinical manifestations, which can influence their hemodynamic responses to exercise.

Previous studies have shown abnormal cardiac and coronary artery functional responses to isometric handgrip (HG) exercise, as well as aortic stiffness and exercise intolerance in HF patients. However, to date, no study has been performed to evaluate the pulmonary artery property changes to isometric exercise in patients with HF^10–12^.

Isometric HG represents a minimal yet effective stressor of the cardiovascular system, and is therefore well-suited for evaluating cardiovascular function in HF patients who may not tolerate maximal exercise^13^. Anomalous cardiovascular responses to HG exercise could signify early alterations in PA compliance, thereby providing valuable diagnostic and prognostic insights^13,14^.

Current methodologies for evaluating PA compliance include right-heart catheterization as the reference standard method for hemodynamic assessments^15,16^. Echocardiographic attempts to assess PA stiffness and hemodynamic flow parameters are limited by frequent suboptimal acoustic windows. Cardiovascular magnetic resonance (CMR) is a comprehensive, noninvasive technique for evaluating both PA compliance and flow hemodynamics^16^.

In light of these considerations, the present study aims to (1) employ advanced CMR techniques to examine changes in PA compliance in response to HG exercise in both healthy subjects and HF patients, (2) elucidate the relationship between dynamic shifts in PA compliance and exercise-induced changes in PA hemodynamics, and (3) compare findings from healthy controls to patients with HF.

By focusing on PA compliance and incorporating isometric HG exercise with advanced CMR techniques, this study seeks to provide novel insights into the pathophysiological underpinnings of HF, potentially informing both clinical diagnosis and management strategies.

## Methods

### Study population

This study was previously reported as a prospective study conducted at two centers in Berlin, Germany, namely at the Charité — University Medicine Berlin and the German Heart Centre Berlin, between 2017 and 2018^14,17–20^.

Briefly, the study cohort comprised individuals aged 45 years or older, who had a confirmed diagnosis of heart failure (HF) NYHA functional Classes II and III established a minimum of 30 days prior to the commencement of the study^17^. All participants were in a stable phase of their respective condition, exhibiting no alterations in their prescribed HF medications and with no hospital admissions related to HF within the week preceding the study. The participants were further categorized based on left ventricular ejection fraction (LVEF) and NT-proBNP levels. Those with LVEF < 40% were classified as having heart failure with reduced ejection fraction (HFrEF). Subjects with LVEF ranging ≥ 40% and < 50% and an elevated NT-proBNP level (> 220 pg/mL) were categorized as having heart failure with mid-range ejection fraction (HFmrEF)^17^. Lastly, individuals presenting an LVEF of 50% or more and an elevated NT-proBNP level were determined to have heart failure with preserved ejection fraction (HFpEF)^17^. For the purpose of participant recruitment, the specific causes of HF were not differentiated^17^. We also enrolled individuals who did not have heart failure or severe cardiovascular ailments to serve as a control group. Subjects who did not complete the study protocol were excluded from the analysis.

Our research adhered strictly to the principles laid out in the Declaration of Helsinki. The respective ethical review board granted approval for the research protocols (Charité – ethics approval no. EA4/112/16). The written informed consent was obtained from all participants for study participation. The study was duly registered with the German Clinical Trials Register (DRKS) under the registration number DRKS00015615. Comprehensive information regarding the criteria for inclusion and exclusion can be accessed on the DRKS website.

### Study protocol

The population analyzed is a subpopulation of the study population characterized by the completion of the examinations required. As previously described, all subjects recruited underwent a comprehensive clinical work-up, which included physical examination, laboratory evaluation, ECG, and a 6-min walk test (6MWT) for the evaluation of functional capacity. Pertinent information such as medical history, current diagnoses, and medication regimen were extracted from electronic health records as described in our previous analyses^9,11,12,17,21^.

All CMR examinations were performed using a 1.5 T MR scanner (Achieva, Philips, Best, the Netherlands) with a phased-array cardiac coil and retrospective ECG gating.

**Figure 1:**
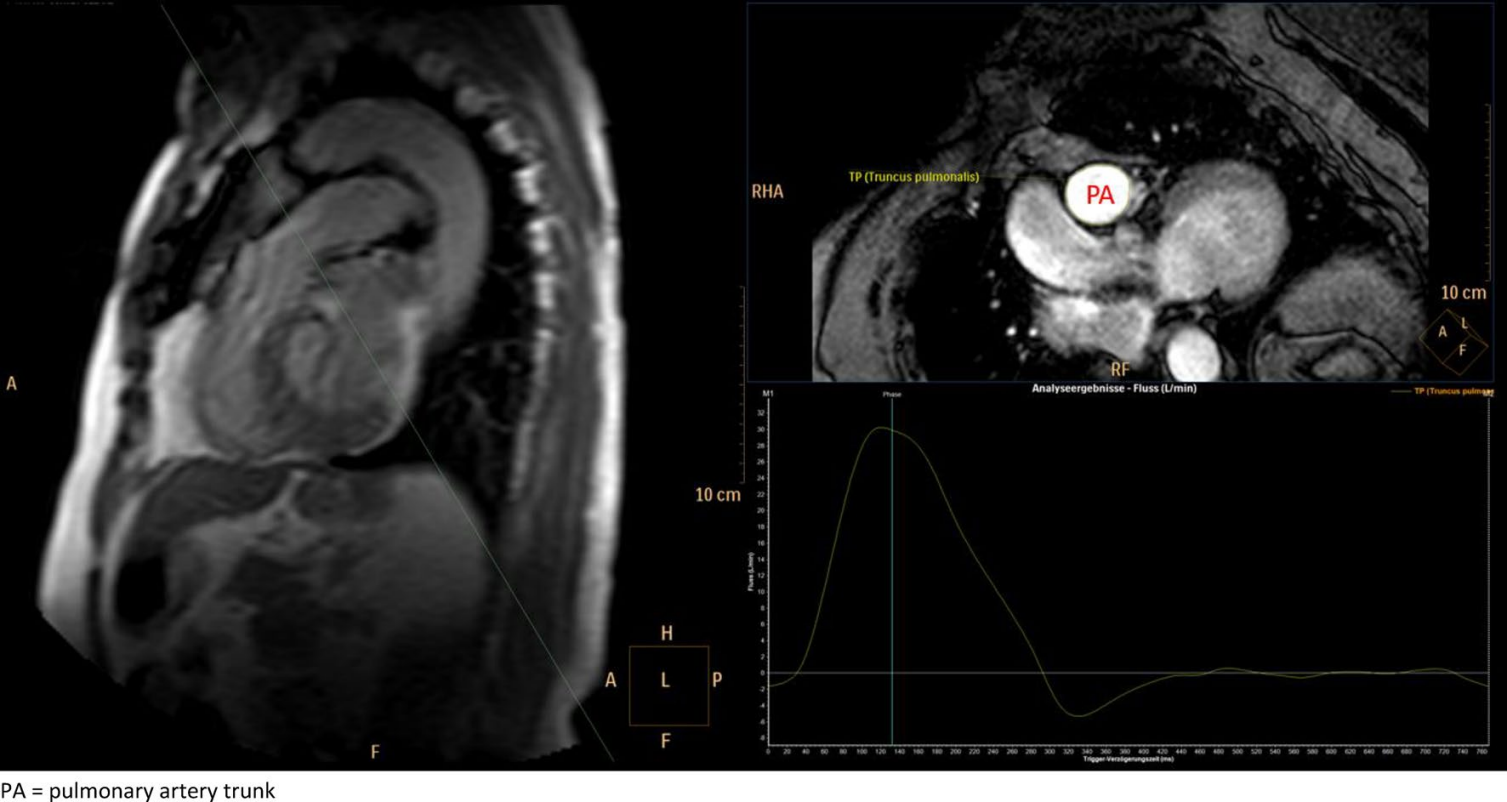
Example of a cross-sectional image of the PA using a phase-contrast through-plane sequence. PA, pulmonary artery trunk.

Cine images were acquired in both short-axis and long-axis orientations during 10–15 s breath-holds, employing a balanced steady-state free precession sequence to measure LVEF. Cross-sectional images of the PA were obtained for vascular quantification using phase-contrast through-plane sequences perpendicular to the pulmonary artery trunk (Fig. 1). The assessment included three short-axis planes (apical, mid, and basal level) as well as two-, three-, and four-chamber planes.

For isometric HG exercise testing, an MRI-safe hand dynamometer (Stoelting, Wood Dale, Illinois) was used. After determining the maximum voluntary contraction using the dominant hand, subjects were instructed to maintain 30% of the maximum voluntary contraction for approximately 3 min, while avoiding Valsalva maneuver by continuous breathing. During the HG exercise, each participant’s heart rate and brachial artery blood pressure, including both systolic and diastolic values, were recorded. From these, pulse pressure was calculated as *PP* = *systolic BP—diastolic BP*, and the rate-pressure product (RPP) was computed as *RPP* = *systolic BP x heart rate*.

### CMR image analysis

CMR images were analysed offline at our CMR-core lab using Medis Suite (version 3.1, Leiden, the Netherlands). LV contours were outlined manually on the cardiac cine images using QMass 8.1. Contours of the main PA were semiautomatically traced and manually adjusted where needed for each phase using QFlow 8.1. PA maximum cross-sectional area (A_max_) and minimum area (A_min_) were acquired in end-systolic and end-diastolic phase images, respectively.

### Assessment of pulmonary artery flow hemodynamics

The following PA blood flow indices, including net flow volume (NFV), flow per min (FPM), peak pressure gradient (PPG) and peak flow velocity (PV), were obtained after drawing vessel contours.

### Determination of pulmonary artery stiffness

The main compliance parameter analyzed to assess PA stiffness, in line with our previous analysis, was the relative area change (RAC) based on the PA area change (AC) in two stages: at rest and during stress testing. The AC was defined by the maximum area (A_max_) and the minimum area (A_min_) as AC = A_max_−A_min_, RAC was defined respectively as RAC = (A_max_−A_min_)/A_min_. A smaller area change (both AC and RAC) indicates a stiffer vessel^22,23^.

PA pulse wave velocity (PWV) was evaluated using the flow area method and calculated as the ratio between flow variation (Δflow) and area variation (Δarea). The PWV values were derived from the slope of a line fitted to the early systole. Acceleration time (AT) and ejection time (ET) were determined as the time interval between the onset and peak blood flow and the time interval between the onset and end of systolic blood flow, respectively^23^.

### Statistical analysis

Values for categorical variables are represented as percentages, while values for continuous variables are given as the mean ± standard deviation. The normality of continuous variables was assessed using the Shapiro–Wilk test. For variables that did not follow a normal distribution, we applied logarithmic transformation to approximate normality. If normality was still not achieved post-transformation, non-parametric tests such as the Wilcoxon signed-rank test were used for paired comparisons instead of the paired t-test. For normally distributed data, a paired t-test was employed to examine differences between baseline and stress measurements. Correlations between variables were tested by Pearson correlation coefficients, and linear regression analysis was applied for a more comprehensive evaluation of these relationships. The intraobserver and interobserver variability in flow measurements were assessed in a sample of 10 subjects (comprising 5 controls and 5 patients) by Bland–Altman analysis (which provided the mean difference and the 95% confidence interval). Statistical packages used to carry out these analyses included SPSS 26.0 (based in Chicago, USA), GraphPad Prism 8.2 (San Diego, USA), and MedCalc 19.0 (Belgium).

## Results

### Population characteristics

Of the 74 subjects prospectively enrolled for the study, 36 successfully completed a HG test as the others did not consent to use the HG device while in the scanner. This finally analyzed population included 19 control subjects and 17 patients who have been diagnosed with HF. The distribution of HF types within the analyzed HF group was 29.41% HFrEF (n = 5), 29.41% HFmrEF (n = 5), and 41.18% HFpEF (n = 7) patients. Baseline characteristics of the analyzed subpopulation, patients of the final dataset for this analysis, are presented in Table 1.

**Table 1:** Baseline characteristics of the analyzed cohort.

Parameter	Controls (n = 19)	HF patients (n = 17)	*p* value
Age (years)	62.0 ± 8.1	68.2 ± 11.9	0.06
Male, n (%)	10 (53)	14 (82)	0.26
BSA (m^2^)	1.86 ± 0.24	1.96 ± 0.14	0.17
NYHA functional class II, n (%)	0 (0)	14 (82)	< *0.01*
NYHA functional class III, n (%)	0 (0)	3 (18)	0.10
6MWD (m)	522.9 ± 118.5	406.4 ± 71.2	< *0.01*
HFrEF, n (%)		5 (29)	
HFmrEF, n (%)		5 (29)	
HFpEF, n (%)		7 (41)	
Blood explorations			
CRP (mg/l)	1.27 ± 1.23	1.97 ± 1.41	0.15
NT-proBNP (ng/l)	88.7 ± 61.1	430.2 ± 298.7	< *0.01*
Echocardiography			
E/e′	7.9 ± 2.2	17.0 ± 5.7	< *0.01*
Cardiac MRI			
LVEF (%)	63.9 ± 4.6	47.1 ± 12.7	< *0.01*
Comorbidities			
Smoking, n (%)	0 (0)	9 (53)	< *0.01*
Arterial hypertension, n (%)	0 (0)	11 (65)	< *0.01*
Hypercholesterolemia, n (%)	0 (0)	8 (47)	< *0.01*
Diabetes mellitus, n (%)	0 (0)	4 (24)	*0.00*
Stroke, n (%)	0 (0)	1 (6)	0.47
COPD, n (%)	0 (0)	1 (6)	0.47
CAD, n (%)	0 (0)	11 (65)	< *0.01*
Previous MI, n (%)	0 (0)	2 (12)	0.22
Medication			
Diuretic, n (%)	0 (0)	5 (29)	*0.02*
ACE inhibitors, n (%)	0 (0)	6 (35)	< *0.01*
ARB, n (%)	0 (0)	8 (47)	< *0.01*
Calcium channel blockers, n (%)	0 (0)	1 (6)	0.47
β-BIockers, n (%)	0 (0)	12 (71)	< *0.01*

6MWD, 6 min walk distance; ACE, angiotensin converting enzyme; ARB, angiotensin receptor blocker; BMI, body mass index; CAD, coronary artery disease; COPD, chronic obstructive pulmonary disease; CRP, C-reactive protein; E, early diastolic peak (pulsed-wave Doppler); e′, early diastolic mitral annular velocity by Doppler tissue imaging; HFmrEF, heart failure with mid-range ejection fraction; HFpEF, heart failure with preserved ejection fraction; HFrEF, heart failure with reduced ejection fraction; LVEF, left ventricular ejection fraction; MI, myocardial infarction; NT-proBNP, N-terminal pro brain natriuretic peptide; NYHA, New York Heart Association.

Significant values are Italic.

### Resting state assessment: PA stiffness, cross-sectional area, and blood flow parameters

**Figure 2:**
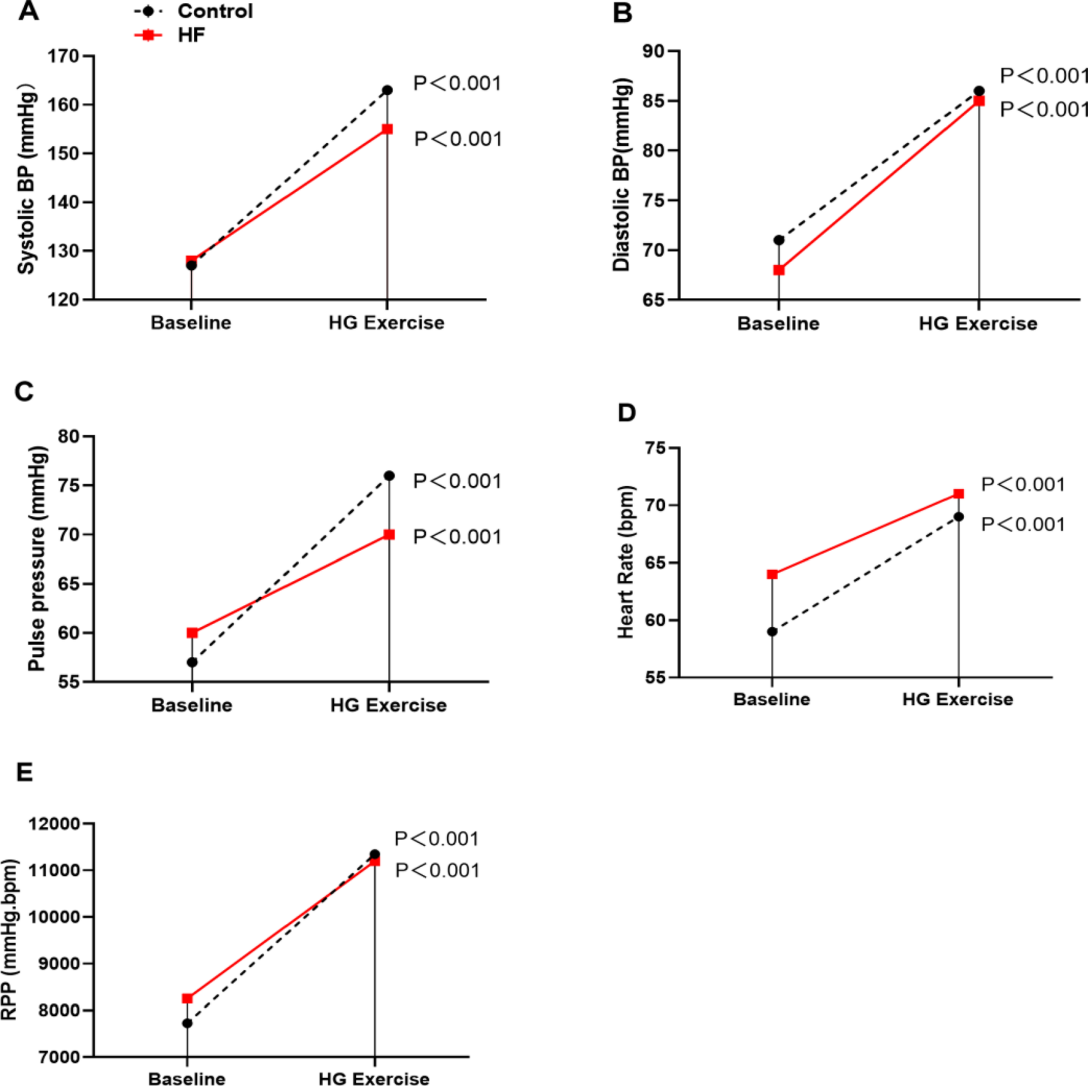
Central hemodynamic response to isometric handgrip exercise. Changes in (**A**) systolic BP, (**B**) diastolic BP, (**C**) pulse pressure, (**D**) heart rate, and (**E**) RPP in healthy controls and HF patients. Black dashed lines represent the healthy control group, whereas red solid lines represent the HF group. BP = blood pressure; HF = heart failure; RPP = rate pressure product.

Both groups exhibited similar levels of systolic BP, diastolic BP, pulse pressure, HR, and RPP at rest (Table 2 and Fig. 2). However, HF patients demonstrated a larger PA diameter at rest, denoted by significantly higher A_min_, and showed diminished PA compliance, indicated by lower AC, RAC, and AT values and higher PWV values than healthy controls (Table T1, supplementary material).

**Table 2:** Central hemodynamics at rest and during handgrip exercise.

Parameter	Controls (n = 19)	HF patients (n = 17)	*p* value
Systolic BP (mmHg)			
Rest	126.8 ± 9.6	125.9 ± 22.8	0.83
HG exercise	163 ± 20*	155 ± 21*	0.24
% change	27 ± 13	22 ± 14	0.33
Diastolic BP (mmHg)			
Rest	71.1 ± 6.3	67.8 ± 8.9	0.28
HG exercise	86 ± 8*	85 ± 8*	0.52
% change	22 ± 9	25 ± 14	0.41
Pulse pressure (mmHg)			
Rest	55.7 ± 8.5	60 ± 17	0.37
HG exercise	76 ± 18*	70 ± 17*	0.28
% change	33 ± 22	19 ± 18	*0.04*
HR (bpm)			
Rest	60.0 ± 8.6	64.9 ± 10.4	0.17
HG exercise	69 ± 10*	71 ± 10*	0.46
% change	16 ± 11	12 ± 7	0.11
RPP (mmHg bpm)			
Rest	7728 ± 1450	8251 ± 2142	0.43
HG exercise	11347 ± 2374*	11,196 ± 2,562*	0.86
% change	48 ± 26	37 ± 18	0.18

*Significant difference between rest and HG (*p* < 0.001), assessed with paired t-test.

BP, blood pressure; HG, handgrip; HR, heart rate; RPP, rate pressure product.

However, HF patients demonstrated increased PA diameter at rest, as highlighted by significantly higher A_min_, and a diminished PA compliance indicated by lower AC, RAC, and AT values and higher PWV values than healthy controls (Table T1, supplementary material). Specifically, the RAC at baseline was notably lower in HF patients (28.9%) compared to controls (50.1%), representing a 21.2% difference and confirming an initial elevated state of PA stiffness in the HF group.

### Exercise-induced changes: hemodynamic responses, PA stiffness, and blood flow

Isometric HG exercise induced a significant increase in systolic BP, diastolic BP, pulse pressure, HR, and RPP in both groups (Table 2 and Fig. 2).

#### Exercise-induced changes in PA cross-sectional area and stiffness

The changes in PA compliance parameters were more muted in HF patients compared to healthy controls during the HG test. The HG exercise stress resulted in an increase in A_max_, although this was not statistically significant in either group (controls: 798.9 ± 201.3 at rest and 800.3 ± 191.0 during HG test, *p* = 0.94 and HF patients: 804.3 ± 160.0 at rest and 792.8 ± 194.7, *p* = 0.58, during HG test). However, the increase in A_min_ was significant in the control but not the HF group (controls: 511.2 ± 124.3 at rest and 591.1 ± 148.6, *p* < 0.01, during HG test and HF patients: 638.3 ± 127.8 at rest and 619.8 ± 157.9, *p* = 0.27, during HG test), corroborating that HF patients have less room to adapt their PA compliance under stress. (Table T3, Figure S1, supplementary material).

#### Exercise-induced changes in PA blood flow parameters

HG exercise stress led to an increase in PA blood flow parameters (NFV, FPM, PPG, and PV) in both groups (Table T1, supplementary material) which was, however, not significant except for the increase in FPM.

### Associations between PA stiffness, hemodynamics, and clinical indicators

#### Correlation with NYHA class and NTproBNP level

Table T2 (supplementary material) delineates the relationships of NT-proBNP levels and NYHA class with PA parameters. Both, NT-proBNP and NYHA class values, showed associations with both resting and exercise-induced changes in PA stiffness and compliance (assessed by PWV, AC, RAC, PWV, ET, and NFV).

#### Correlation with functional capacity (6MWD)

**Figure 3:**
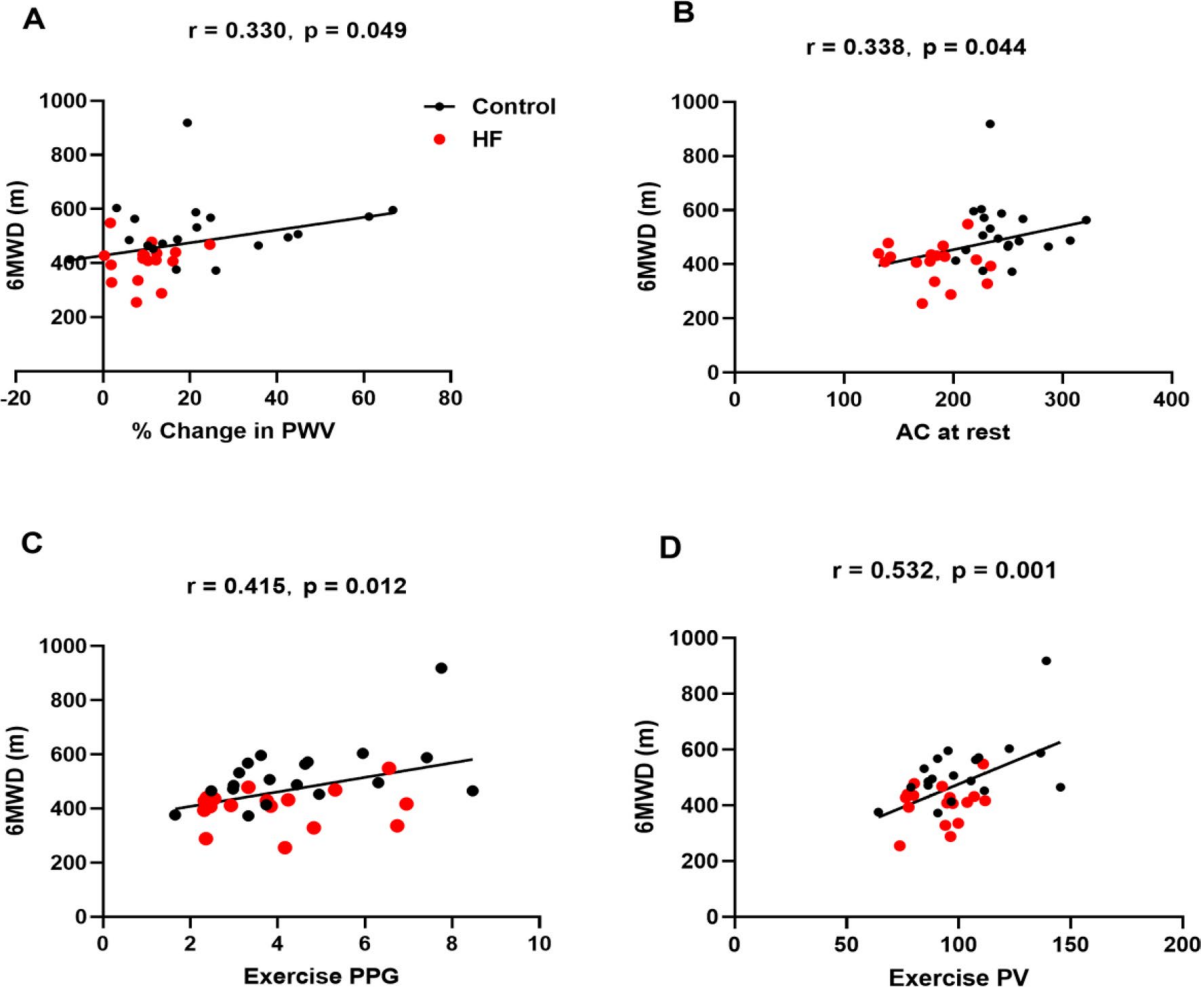
Correlation model for PA compliance and functional capacity (6MWD). Assessment of the change in PWV (**A**), AC at rest (**B**), exercise PPG (**C**), and exercise PV (**D**). All correlations reported are Pearson correlation coefficients. Black solid lines represent the fitted linear regression curve for all datasets. 6MWD = 6 min walk distance; AC = area change; PPG = peak pressure gradient; PV = peak flow velocity; PWV = pulse wave velocity.

Figure 3 illustrates the significant associations between functional capacity and PA parameters both at rest and at HG. Significant correlations were observed between functional capacity, measured as 6MWD, and PA parameters both at rest and during HG exercise (assessed by PA PPG, PA PV, and PA PWV).

#### Correlation of pulmonary distensibility parameters with NTproBNP levels

**Figure 4:**
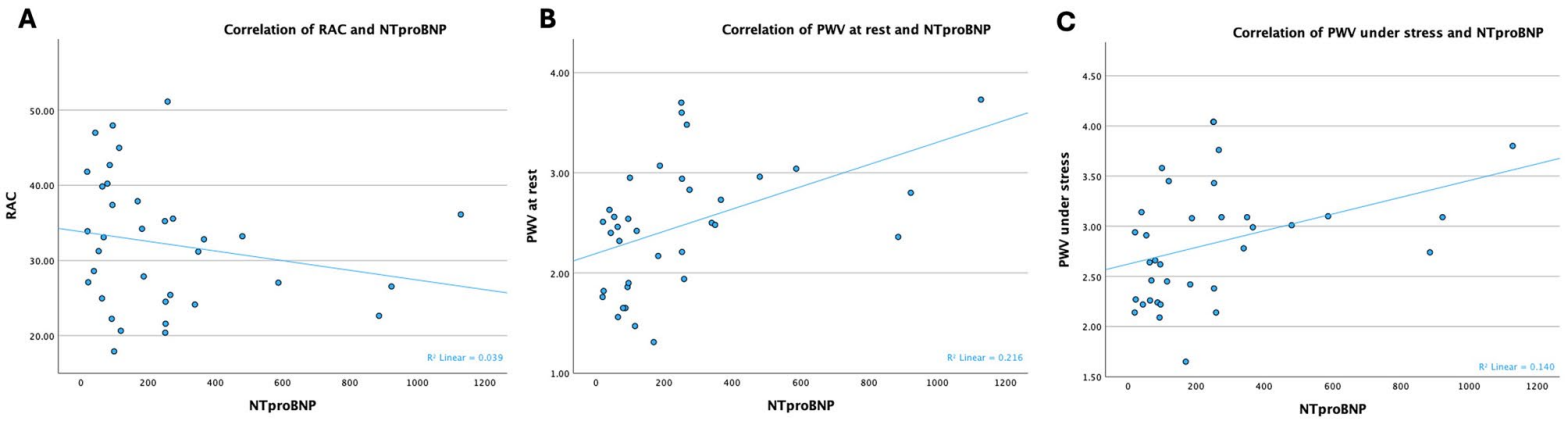
Correlation of pulmonary distensibility parameters with NTproBNP levels. Correlation of relative area change (RAC) as well as pulmonary artery pulse wave velocity (PWV) at rest and under stress with NTproBNP. (**A**) Correlation of RAC with NTproBNP: r = -0.198, *p* = 0.253; (**B**) Correlation PWV at rest with NTproBNP: r = 0.465, *p* = *0.005* (significance level 0.01); (**C**) Correlation PWV under stress with NTproBNP: r = 0.374, *p* = *0.027* (significance level 0.01).

Figure 4 shows the intricate relationships between pulmonary distensibility parameters and NTproBNP levels both at rest and during HG. An inverse association between RAC and NTproBNP levels is suggested, while not significantly correlated. Furthermore, there is a significant positive correlation between PWV at rest and NTproBNP levels. Likewise, during HG stress, the PWV showcases a significant positive relationship with NTproBNP levels.

### Reproducibility and interobserver variability in CMR measurements

Bland–Altman analysis indicated low intra- and interobserver variability in CMR measurements (Table T3 & Figure S2, supplementary material).

## Discussion

In this analysis, we explored PA hemodynamic alterations in individuals with stable HF relative to healthy controls utilizing isometric HG exercise stress and CMR.

The results shed light on three primary areas:

1. HF patients show significantly larger PA diameter at rest compared to controls, suggesting higher PA congestion.
2. HF patients show a smaller reaction in PA compliance parameters under HG exercise, as they already start with a pre-dilated PA system.
3. A correlation between CMR-assessed PA hemodynamics and functional capacity (6MWD).

This study describes for the first time the effects of HG exercise on non-invasively assessed PA compliance and blood flow using advanced CMR in HF patients and controls PA compliance, often referred to by its inverse antonym *PA stiffness*, is a key metric that describes the ability of the pulmonary artery to expand and contract, and is not merely a passive tissue property. Similar to previous research, also analysis from our group, our results demonstrated a significant increase in HR, RPP, systolic BP, diastolic BP, and pulse pressure during isometric HG exercise in both healthy controls and HF patients^14,24^.

An essential finding from our study is the significantly reduced PA compliance in HF patients. The increase in PA compliance parameters, or the increased PA stiffness is higher respectively, during exertion is smaller in HF patients than in controls during the HG test. This underlines the impaired PA vascular reactions to exercise. Given that HF patients start with a pre-dilated PA system, they have less room to adapt under stress, potentially resulting in a muted response in PA compliance. This suggests that it might not just be the absolute levels of compliance but the dynamic change in compliance during exercise that could have clinical significance.

We hypothesize, and future studies should validate, that increased baseline congestion due to HF limits the chance to adapt the PA system and further extend its diameter. Increased PA stiffness leads to higher pulmonary vascular resistance, increasing the afterload for the right ventricle. This difficulty is aggravated during exercise, resulting in fatigue, breathlessness, and ultimately, exercise intolerance^25–27^.

The change in heart rate was not different between the groups in our study, which aligns with the findings of previous studies and other analysis on the same subjects by our group indicating a surge in HR, RPP, systolic BP, diastolic BP, and pulse pressure during isometric HG exercise among both healthy controls and HF patients^14,28–31^.

Our findings of a larger PA diameter at rest and a muted increase in PA compliance during HG exercise in HF patients offer new insights into their abnormal vascular responses to physical stress. In this context, we hypothesize that pre-dilated PA systems in HF patients limit their vascular adaptability during exercise compared to controls. This supports the notion that increased baseline PA congestion due to HF hampers the capacity to further dilate the PA system during exercise. Although associations between PA congestion and PA coupling in HF have previously been described, they but not been tracked non-invasively by CMR^32^. Furthermore, decreased PA compliance at baseline seems to result in limited flexibility for additional vascular adaptation during exercise, which in turn, could contribute to exercise intolerance in HF patients.

While our study demonstrates significant associations, itis crucial to emphasize that these relationships do not necessarily imply causation. Further studies are required to elucidate the potential causal relationships between these variables, particularly in the context of cardiac pre- and afterload parameters. In larger observational datasets with longitudinal follow-up there is the chance to approach this challenge with statistical methods, e.g. Cox regression models with time-varying covariates that were developed to handle complex longitudinal data and the dual role of dynamic variables as both confounders and mediators, which can bias effect sizes. These methods allow for more accurate estimation of causal effects by appropriately adjusting for time-varying confounders and mediators^33^.

However, it is essential to underline the general benefits of using CMR to assess PA characteristics in these patients as CMR provides high spatial resolution and comprehensive imaging capabilities, allowing for detailed evaluation of both structural and functional aspects of the pulmonary arteries. Unlike invasive methods, CMR is safer and more comfortable for patients, facilitating repeated measurements and longitudinal studies. These advantages enable a more accurate and holistic understanding of PA compliance and hemodynamics, which is essential for advancing HF management and improving patient outcomes.

### Limitations

Our study has limitations that could impact the interpretation of our findings. Firstly, it was a single-center study, introducing the possibility of center-specific bias. The relatively small sample size limits the generalizability of our findings, potentially under-representing the overall population of HF patients. Furthermore, medications were not withdrawn during the study; hence, potential confounding effects of medications cannot be ruled out. The study does not differentiate between the importance of dynamic changes in PA compliance versus the absolute levels reached during exercise, which could have clinical relevance particularly in the setting of exercise intolerance.

Given the heterogeneity of HF, our findings may not fully capture the nuances of each subgroup. Therefore, we propose that future studies with larger cohorts should stratify patients based on specific HF characteristics to better understand the differential impact on PA compliance and overall cardiovascular function^34^. Additionally, we recommend considering right-heart catheterization data to complement non-invasive assessments and provide a more comprehensive understanding of PA hemodynamics in diverse HF populations.

### Perspectives

Furthermore, it would be valuable to investigate whether these observed abnormalities in PA diameter and compliance could serve as a therapeutic target or diagnostic parameters in improving functional capacity and overall quality of life in HF patients, especially in monitoring decongesting techniques.

## Conclusion

In conclusion, this study demonstrated that HF patients exhibit an abnormal PA vascular response to isometric handgrip exercise, characterized by larger PA diameters at rest and smaller changes in PA compliance during exercise, which correlates with functional capacity. Our novel findings and their potential impact on the understanding of HF pathology have potential clinical implications. In terms of future application, these results could potentially be used to guide early interventions to prevent cardiac events in HF patients. Further research is warranted to explore the therapeutic implications of these findings to improve HF management and patient outcomes.

### Supplementary Information

Supplementary Information.

## Data Availability

The data underlying this article cannot be shared publicly for the privacy of individuals that participated in the study. The data will be shared on reasonable request to the corresponding author.
