# Supplementary material for: Reduced dynamic changes in pulmonary artery compliance during isometric handgrip exercise in patients with heart failure
Source: Sci Rep. 2024 Jul 6;14:15594. doi: 10.1038/s41598-024-66194-8 (PMC11227514; doi:10.1038/s41598-024-66194-8)
Supplement: Supplementary file 1 — Supplementary Information. [file 41598_2024_66194_MOESM1_ESM.docx]

**SUPPLEMENTARY MATERIAL**

**TABLES AND FIGURES**

**Tables**

Table T1 – CMR measured indices of pulmonary artery hemodynamics at rest and during handgrip exercise

Table T2 – Correlation between NYHA class or NTproBNP level and PA parameters at rest and during exercise

Table T3 – Reproducibility of percent change in CMR PA measurements

**Figures**

Figure S1 – Change in PA compliance indices

Figure S2 – Intraobserver and interobserver agreement of measurements by Bland-Altman plots.

**Table T1 – CMR measured indices of pulmonary artery hemodynamics at rest and during handgrip exercise**

| **Parameter** | | **Healthy Controls**  **(n=19)** | **HF Patients**  **(n=17)** | **P value** |
| --- | --- | --- | --- | --- |
|  | |  |  |  |
|  |  |  |  |  |
| A_max_ (mm^2^) | Rest | 798.9 ± 201.3 | 804.3 ± 160.0 | 0.92 |
|  | Exercise | 800.3 ± 191.0 | 792.8 ± 194.7 | 0.78 |
| A_min_ (mm^2^) | Rest | 511.2 ± 124.3 | 638.3 ± 127.8 | *< 0.01* |
|  | Exercise | 591.1 ± 148.6 | 619.8 ± 157.9 | 0.69 |
| AC (mm^2^) | Rest | 246.6 ± 31.1 | 171.5 ± 41.2 | *< 0.01* |
|  | Exercise | 209.3 ± 62.4 | 168.7 ± 40.6 | *0.03* |
|  | % Change | - 13.9 ± 29.1 | - 7.0 ± 16.7 | 0.15 |
| RAC (%) | Rest | 50.1 ± 9.0 | 28.9 ± 5.4 | *< 0.01* |
|  | Exercise | 35.9 ± 9.3 | 28.0 ± 5.2 | *< 0.01* |
| PWV (m/s) | Rest | 2.1 ± 0.5 | 2.9 ± 0.5 | *< 0.01* |
|  | Exercise | 2.5 ± 0.5 | 3.2 ± 0.6 | *< 0.01* |
| AT (ms) | Rest | 138.1 ± 29.9 | 130.0 ± 19.1 | *< 0.01* |
|  | Exercise | 113.0 ± 12.9 | 107.4 ± 18.9 | 0.29 |
|  |  |  |  |  |
| ET (ms) | Rest | 371.8 ± 35.3 | 343.7 ± 33.8 | 0.0390 |
|  | Exercise | 345.3 ± 20.6 | 320.3 ± 27.8 | *< 0.01* |
| NFV (ml/cycle) | Rest | 83.0 ± 19.6 | 70.0 ± 10.5 | 0.08 |
|  | Exercise | 83.4 ± 14.5 | 73.3 ± 16.3 | *0.04* |
| FPM (L/min) | Rest | 4.9 ± 0.9 | 5.0 ± 1.2 | 0.43 |
|  | Exercise | 5.4 ± 1.0 | 5.0 ± 1.2 | 0.24 |
| PPG (mmHg) | Rest | 3.9 ± 1.5 | 3.2 ± 1.5 | 0.51 |
|  | Exercise | 4.5 ± 1.9 | 4.0 ± 1.6 | 0.34 |
| PV (cm/s) | Rest | 96.7 ± 16.6 | 91.1 ± 13.5 | 0.25 |
|  | Exercise | 102.0 ± 21.6 | 92.1 ± 13.0 | 0.12 |

*Significant difference between rest and exercise (p < 0.05).

Amax = maximum pulmonary artery cross-sectional area; Amin = minimum pulmonary artery across-sectional area; AC = area change; AT = acceleration time; ET = ejection time; FPM = flow per min; NFV = net flow volume; PPG = peak pressure gradient; PWV = pulse wave velocity; PV = peak flow velocity; RAC = relative area change.

**Table T2** **– Correlation between NYHA class or NTproBNP level and PA parameters at rest and during exercise**

|  | | **NYHA Class** | | **NTproBNP level** | |
| --- | --- | --- | --- | --- | --- |
|  |  | **r** | **P value** | **r** | **P value** |
| **Clinical data** | | | | | |
| Age | | 0.298 | 0.078 | 0.085 | 0.620 |
| BSA | | 0.228 | 0.182 | 0.108 | 0.532 |
| **CMR parameters** | | | | | |
| PWV | Rest | 0.613 | *< 0.001* | 0.465 | *0.004* |
|  | Exercise | 0.489 | *0.002* | 0.374 | *0.025* |
| RAC | Rest | - 0.685 | *< 0.001* | - 0.408 | *0.013* |
|  | Exercise | - 0.446 | *0.006* | - 0.196 | 0.252 |
| AC | Rest | - 0.655 | *< 0.001* | - 0.403 | *0.015* |
|  | Exercise | - 0.312 | 0.064 | - 0.122 | 0.480 |
| AT | Rest | - 0.486 | *0.003* | - 0.313 | 0.063 |
|  | Exercise | - 0.143 | 0.404 | - 0.175 | 0.307 |
| ET | Rest | - 0.163 | 0.341 | - 0.089 | 0.605 |
|  | Exercise | - 0.422 | *0.010* | - 0.035 | 0.839 |
| NFV | Rest | - 0.314 | 0.063 | -0.251 | 0.140 |
|  | Exercise | - 0.357 | *0.032* | -0.217 | 0.204 |
| FPM | Rest | - 0.165 | 0.337 | - 0.205 | 0.230 |
|  | Exercise | - 0.231 | 0.175 | - 0.219 | 0.200 |
| PPG | Rest | - 0.120 | 0.486 | - 0.081 | 0.639 |
|  | Exercise | - 0.165 | 0.336 | - 0.114 | 0.509 |
| PV | Rest | - 0.222 | 0.193 | - 0.182 | 0.283 |
|  | Exercise | - 0.301 | 0.074 | - 0.123 | 0.474 |

AC = area change; AT = acceleration time; ET = ejection time; FPM = flow per min; NFV = net flow volume; PPG = peak pressure gradient; PWV = pulse wave velocity; PV = peak flow velocity; RAC = relative area change.

**Table T3 – Reproducibility of percent change in CMR PA measurements**

| **Parameter** | **Bland-Altman*** | |
| --- | --- | --- |
|  | **Intra-observer** | **Inter-observer** |
| % Change in A_max_ | 0.2 (- 3.1 to 3.5) | - 1.5 (- 8.2 to 5.2) |
| % Change in A_min_ | 0.1 (- 5.7. to 5.8) | - 2.1 (- 10.5 to 6.3) |
| % Change in AC | - 0.0 (- 12.7 to 12.7) | - 2.9 (- 29.9 to 24.2) |
| % Change in RAC | - 0.0 (- 14.0 to 14.0) | - 2.1 (- 30.9 to 26.8) |
| % Change in PWV | 0.2 (- 3.2 to 3.6) | 0.1 (- 5.5 to 5.7) |
| % Change in AT | - 1.4 (- 5.9 to 3.0) | - 2.4 (- 11.1 to 6.3) |
| % Change in ET | - 0.5 (- 3.1 to 2.1) | - 1.1 (- 5.6 to 3.4) |
| % Change in NFV | - 0.20 (- 0.65 to 0.26) | - 0.15 (- 1.22 to 0.91) |
| % Change in FPM | - 0.4 (- 3.1 to 3.9) | 0.1 (- 6.5 to 6.6) |
| % Change in PPG | 1.7 (- 2.8 to 6.3) | 0.5 (- 11.3 to 12.3) |
| % Change in PV | 0.3 (- 2.3 to 3.0) | 0.7 (- 2.2 to 3.7) |

*Mean bias (limits of agreement: ±1.96 standard deviations). Abbreviations see Table 2.

**Figure S1 – Change in PA compliance indices**


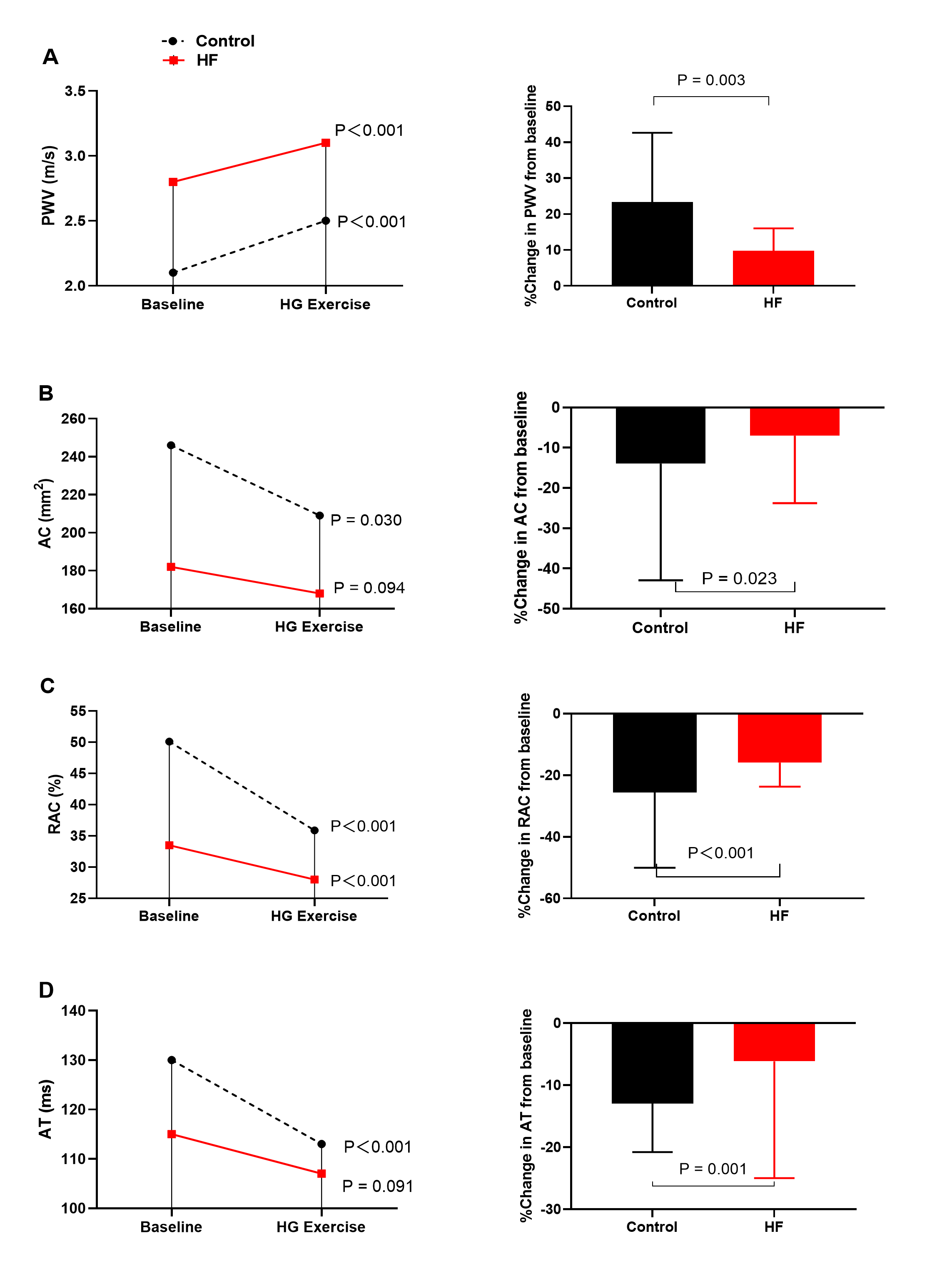


Left panel: Changes in (A) pulse wave velocity (PWV); (B) area change (AC); (C) relative area change (RAC); D: acceleration time (AT) at rest and during handgrip exercise. Right panel: Relative change from baseline in healthy controls and heart failure patients. Black dashed lines represent the control group, whereas red solid lines represent the HF group. Black bars represent the control group, whereas red bars represent the HF group.

**Figure S2 – Intraobserver and interobserver agreement of measurements by Bland-Altman plots.**

A


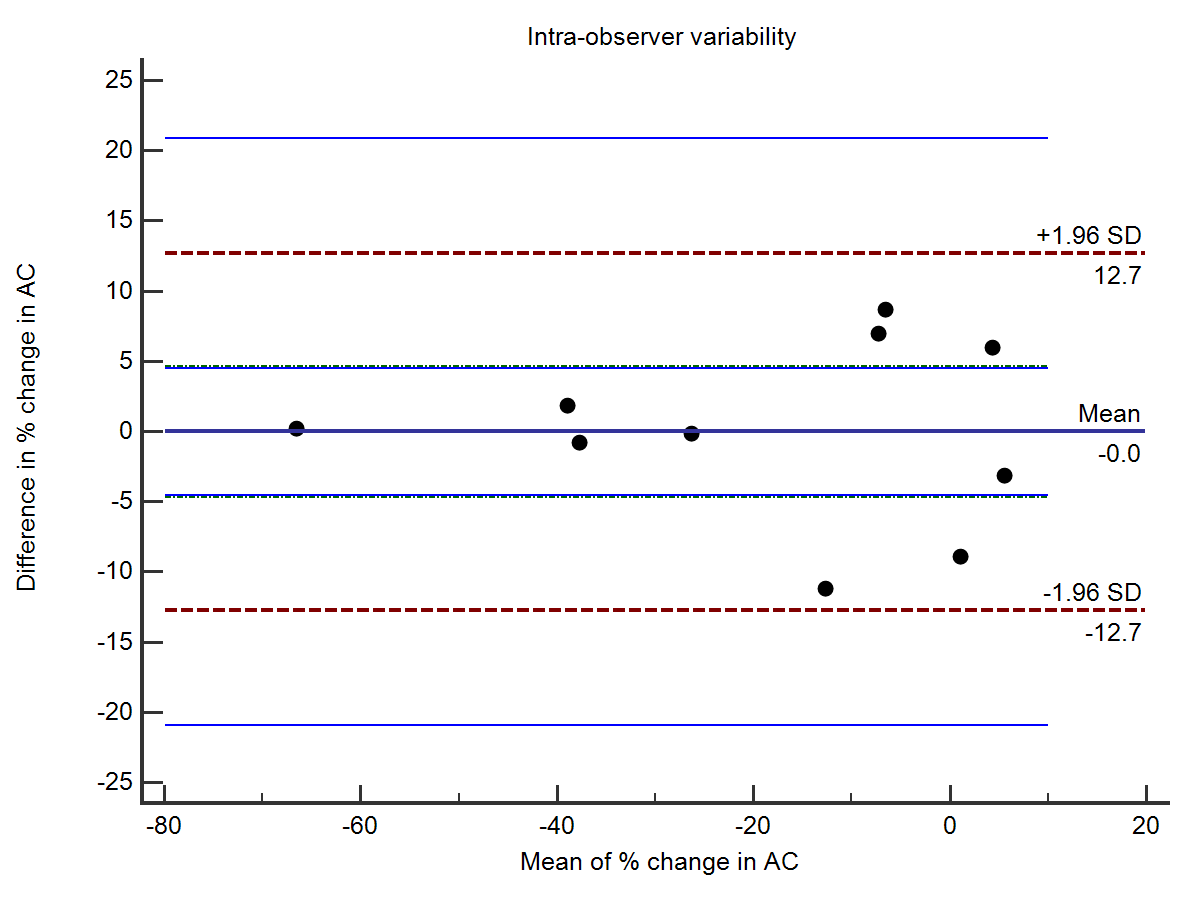

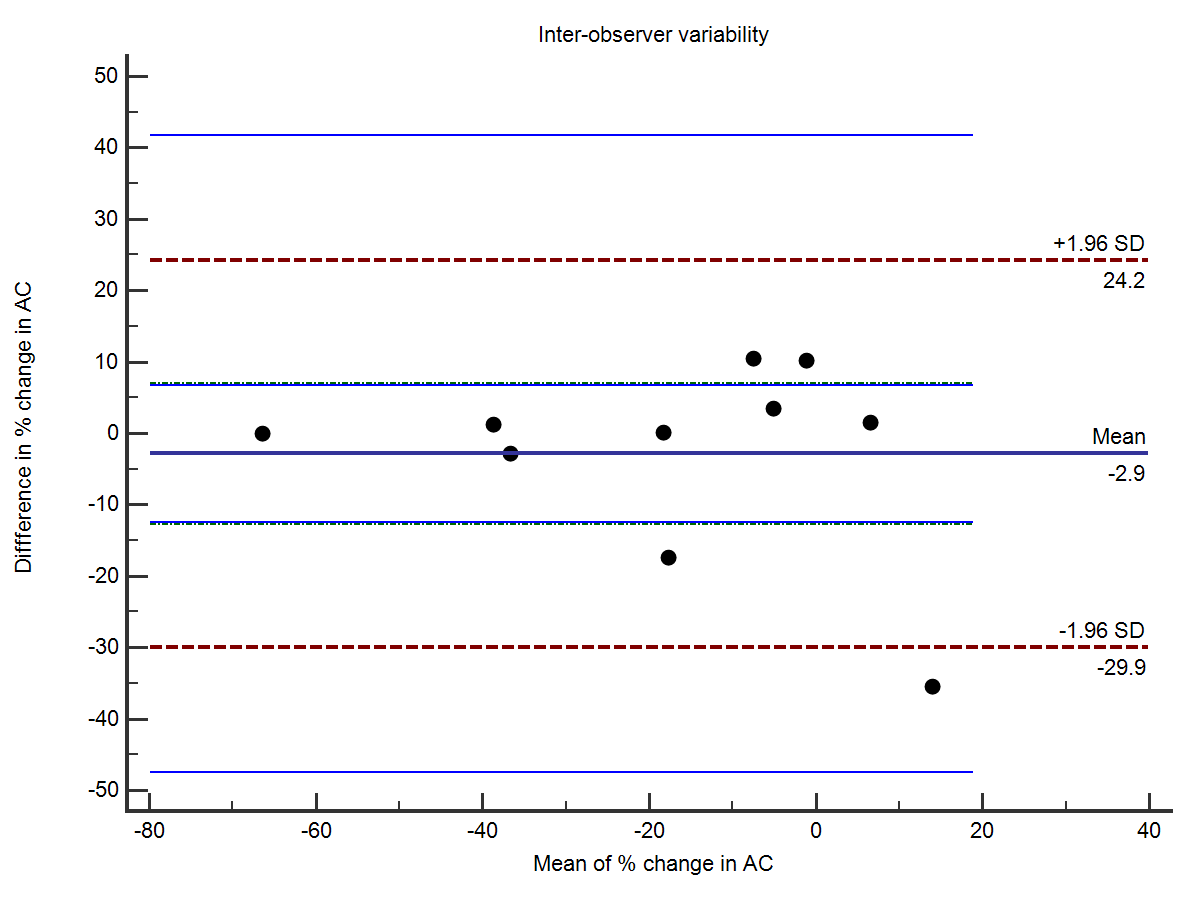


B


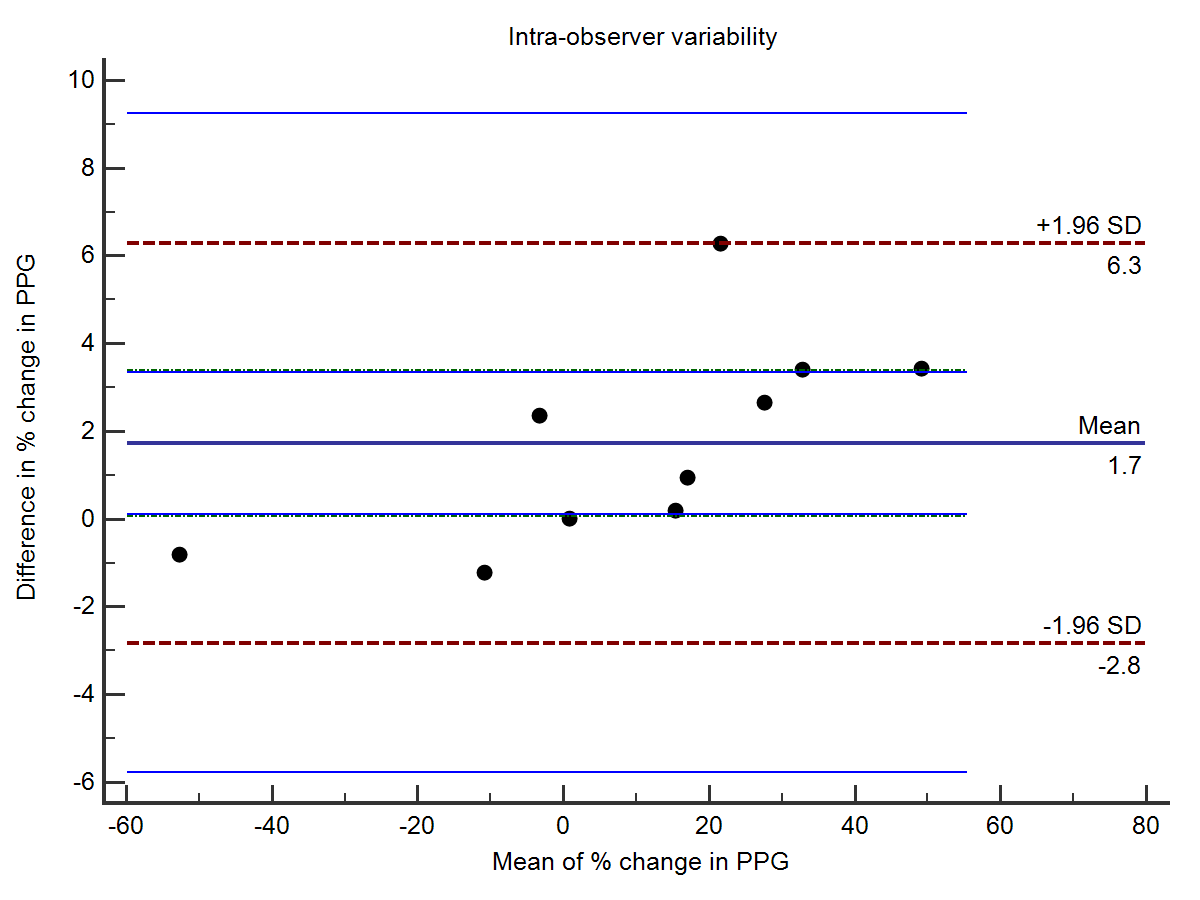

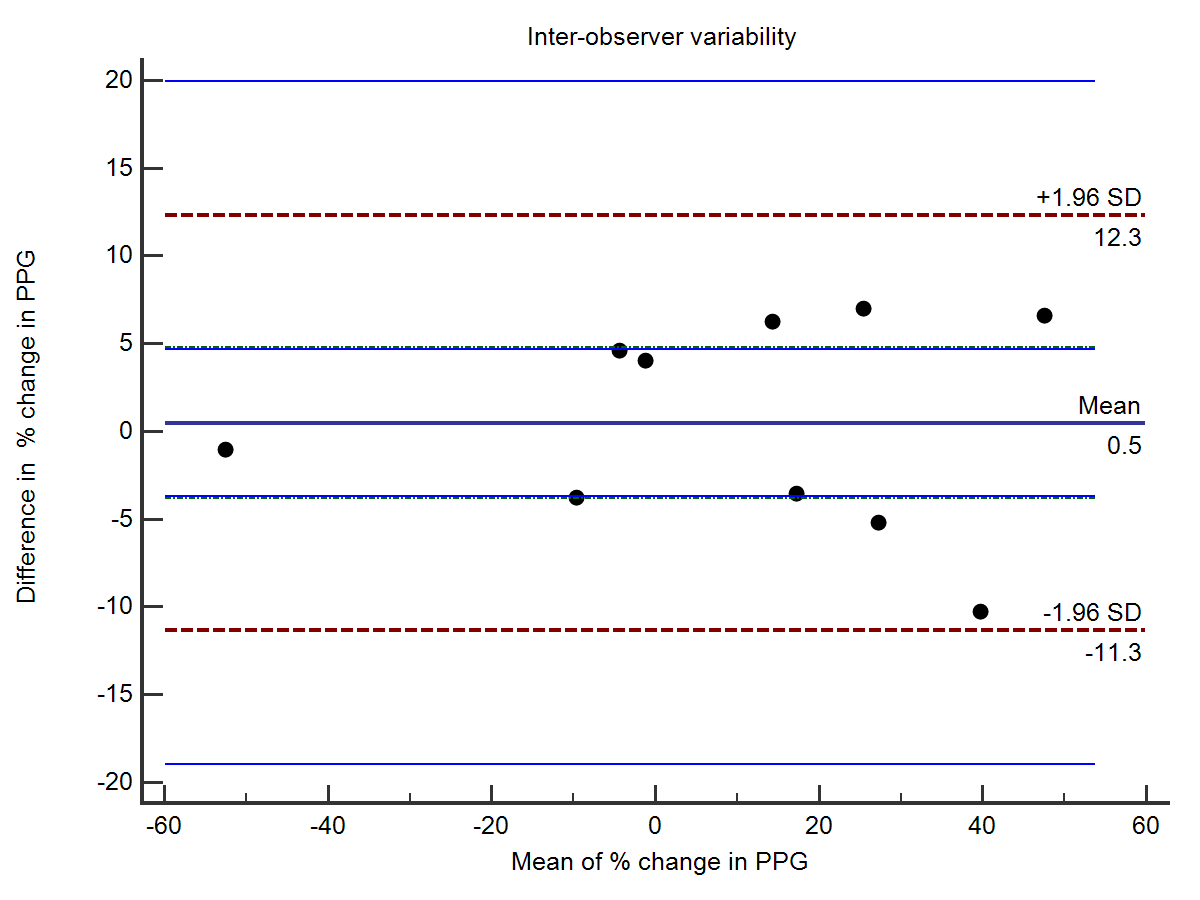


C


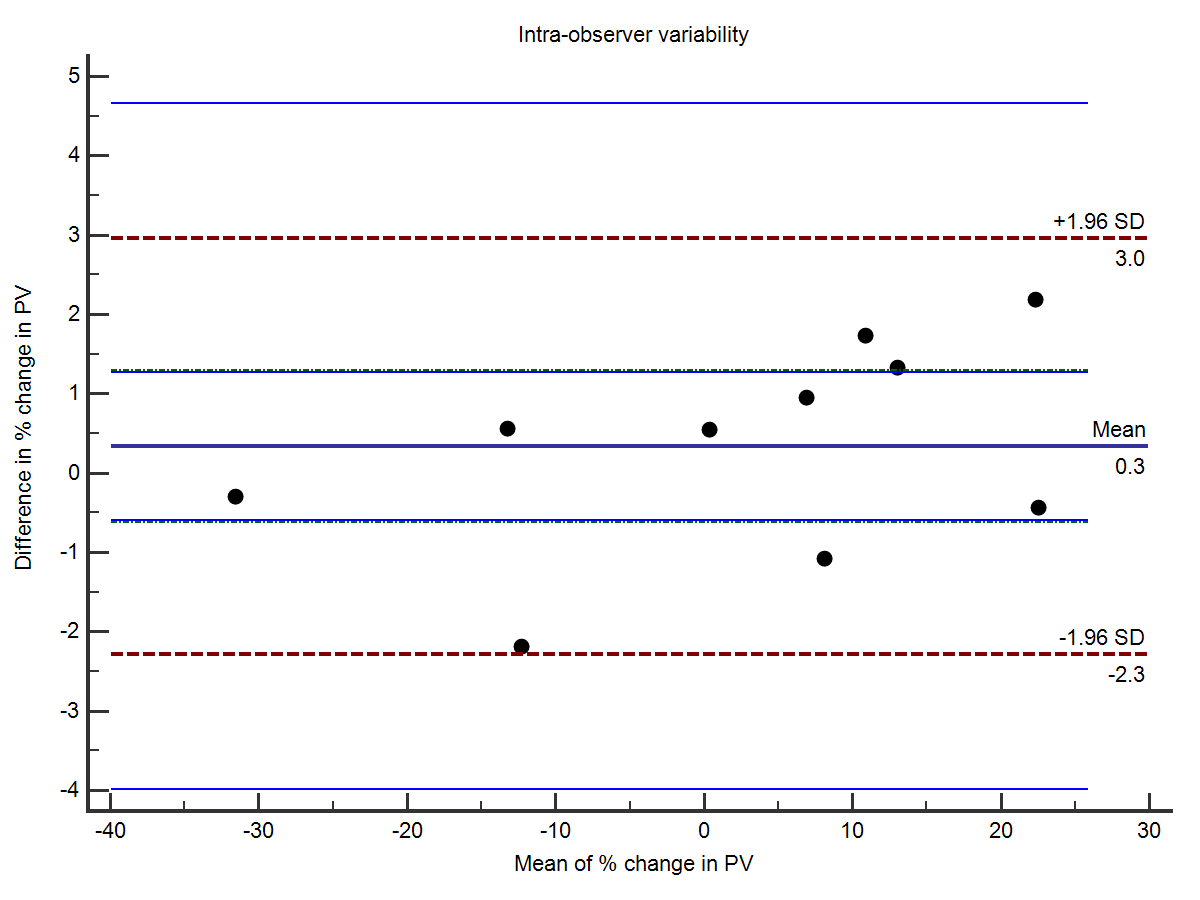

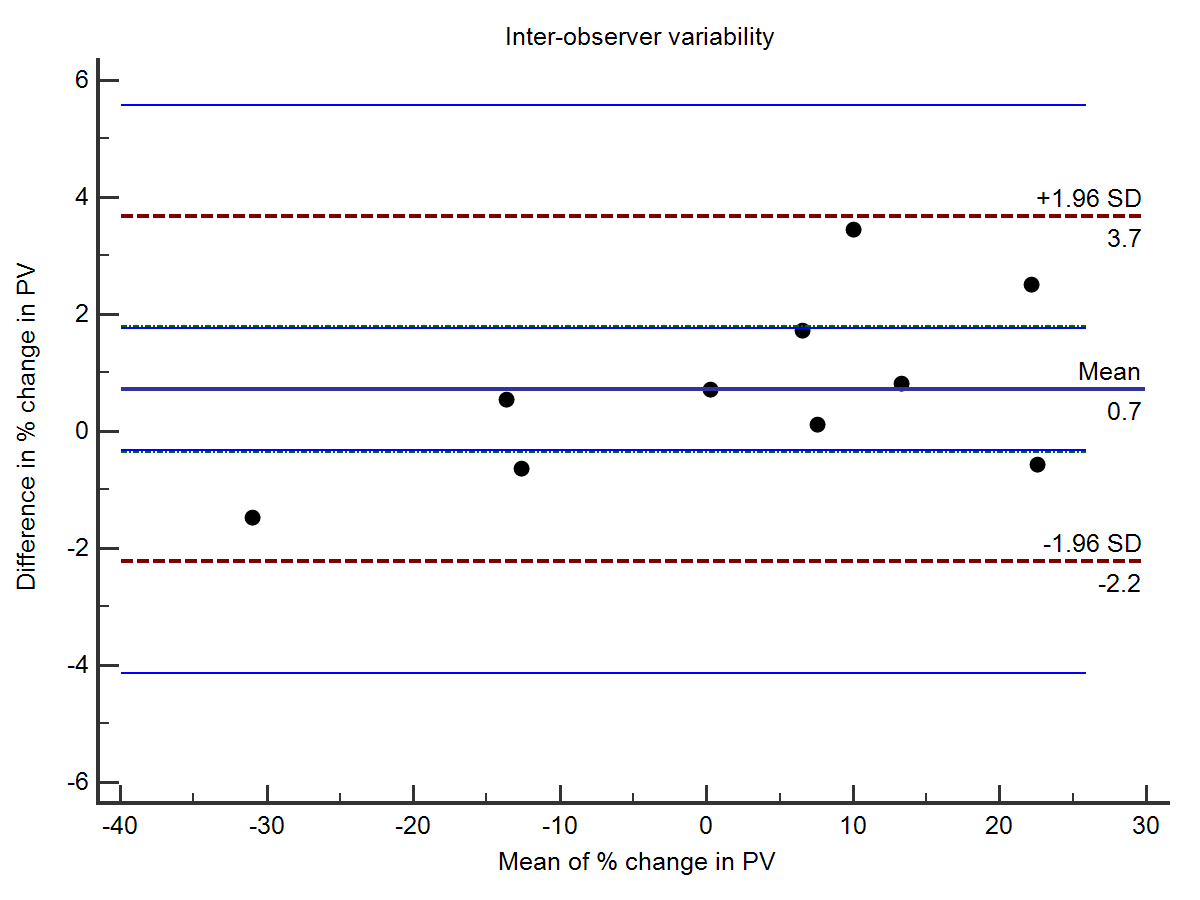


Demonstrating the agreement for percent change in AC (A), PPG (B), and PV (C). The middle-solid line is the mean difference of measures. The upper and lower dashed lines are ±1.96 standard deviation.
